# Patients with non-idiopathic sudden sensorineural hearing loss show hearing improvement more often than patients with idiopathic sudden sensorineural hearing loss

**DOI:** 10.1007/s00405-021-06691-y

**Published:** 2021-03-08

**Authors:** Jovanna Thielker, Anne Heuschkel, Daniel Boeger, Jens Buentzel, Dirk Esser, Kerstin Hoffmann, Peter Jecker, Andreas Mueller, Gerald Radtke, Orlando Guntinas-Lichius

**Affiliations:** 1 Department of Otorhinolaryngology, Jena University Hospital, Am Klinikum 1, 07747 Jena, Germany; 2 Department of Otorhinolaryngology, HELIOS-Klinikum, Erfurt, Germany; 3 Department of Otorhinolaryngology, Zentralklinikum, Suhl, Germany; 4 Department of Otorhinolaryngology, Südharz-Krankenhaus gGmbH, Nordhausen, Germany; 5 Department of Otorhinolaryngology, Sophien/Hufeland-Klinikum, Weimar, Germany; 6 Department of Otorhinolaryngology, Klinikum Bad Salzungen, Bad Salzungen, Germany; 7 Department of Otorhinolaryngology, SRH Wald-Klinikum, Gera, Germany; 8 Department of Otorhinolaryngology, Ilm-Kreis-Kliniken, Arnstadt, Germany

**Keywords:** Idiopathic hearing loss, Non-idiopathic hearing loss, Acute otitis media, Zoster oticus

## Abstract

**Introduction:**

To compare inpatient treated patients with idiopathic (ISSNHL) and non-idiopathic sudden sensorineural hearing loss (NISSNHL) regarding frequency, hearing loss, treatment and outcome.

**Methods:**

All 574 inpatient patients (51% male, median age: 60 years) with ISSNHL and NISSNHL, who were treated in federal state Thuringia in 2011 and 2012, were included retrospectively. Univariate and multivariate statistical analyses were performed.

**Results:**

ISSNHL was diagnosed in 490 patients (85%), NISSNHL in 84 patients (15%). 49% of these cases had hearing loss due to acute otitis media, 37% through varicella-zoster infection or Lyme disease, 10% through Menière disease and 7% due to other reasons. Patients with ISSNHL and NISSNHL showed no difference between age, gender, side of hearing loss, presence of tinnitus or vertigo and their comorbidities. 45% of patients with ISSNHL and 62% with NISSNHL had an outpatient treatment prior to inpatient treatment (*p* < 0.001). The mean interval between onset of hearing loss to inpatient treatment was shorter in ISSNHL (7.7 days) than in NISSNHL (8.9 days; *p* = 0.02). The initial hearing loss of the three most affected frequencies in pure-tone average (3PTAmax) scaled 72.9 dBHL ± 31.3 dBHL in ISSNHL and 67.4 dBHL ± 30.5 dBHL in NISSNHL. In the case of acute otitis media, 3PTAmax (59.7 dBHL ± 24.6 dBHL) was lower than in the case of varicella-zoster infection or Lyme disease (80.11 dBHL ± 34.19 dBHL; *p* = 0.015). Mean absolute hearing gain (Δ3PTAmax_abs_) was 8.1 dB ± 18.8 dB in patients with ISSNHL, and not different in NISSNHL patients with 10.2 dB ± 17.6 dB. A Δ3PTAmax_abs_ ≥ 10 dB was reached in 34.3% of the patients with ISSNHL and to a significantly higher rate of 48.8% in NISSNHL patients (*p* = 0.011).

**Conclusions:**

ISSNHL and NISSNHL show no relevant baseline differences. ISSNHL tends to have a higher initial hearing loss. NISSHNL shows a better outcome than ISSNHL.

**Supplementary Information:**

The online version contains supplementary material available at 10.1007/s00405-021-06691-y.

## Introduction

So far, many studies have analyzed epidemiological data for idiopathic sudden sensorineural hearing loss (ISSNHL) [1, 2]. In a previous study on the ISSNHL, left side, non-declining audiogram type and no previous outpatient treatment as independent prognostic factors for a better recovery could be found [3]. Profound hearing loss, hearing loss in older patients, delayed treatment and arterial hypertension were negative prognostic factors in another study [4]. In sum, there are high reported recovery rates up to 32% to 65% [5, 6]. There are some uncertainties due to therapy strategies but the possible therapy strategies are discussed extensively in therapy recommendations [7]. Systemic glucocorticoid, rheological therapy, local glucocorticoid therapy or even a wait-and-see strategy is currently recommended [8].

In contrast, not much is known on the outcome of non-idiopathic sudden sensorineural hearing loss (NISSNHL), because in all studies analyzing hearing loss and recovery these patients are excluded [9, 10]. The aim of this work is therefore to define whether an underlying cause of hearing loss in patients with NISSNHL is associated with a different prognosis for hearing gain than is the case with ISSNHL.

In the federal state of Thuringia, there are eight hospitals with departments for ears-nose-throat (ENT) medicine. These have formed a network for the scientific evaluation of ENT diseases [11, 12]. Recently, we have published data on all patients who were hospitalized in Thuringia in 2011 and 2012 for treatment of ISSNHL [3, 11, 12]. Here, we compare now the results of these patients with ISSNHL to the patients treated for NISSNHL in the same time period.

## Methods

### Study design and patients

We performed a retrospective analysis in all eight ENT departments of the federal state Thuringia. All patients who were hospitalized in 2011 and 2012 due to acute hearing loss with the ICD codes (International Classification of Diseases) H91.0, H91.1, H91.2, H91.3, H91.8 and H91.9 were included in the study. A positive ethical vote for the evaluation of the underlying data was obtained (No. 2726-12/09, 4755-0416). A total of 723 patients with the above-mentioned diagnoses were treated as inpatient patients and were included in the primary dataset. It made no difference whether the patients had comorbidities or had already received prior outpatient treatment. 58 patients were initially excluded from the evaluation due to missing data sets (e.g. no initial hearing investigation) and 91 patients were excluded due to a lack of initial hearing loss, inpatient treatment due to middle ear surgery or cochlear implantation or lack of follow-up hearing examinations. Of the remaining 574 patients, 490 had an idiopathic sudden sensorineural hearing loss (ISSNHL) while the remaining 84 patients had a disease underlying the hearing loss (Menière disease, acute otitis media, Lyme disease, varicella-zoster infection) and were classified as NISSNHL. All 574 patients, ISSNHL as well as NISSNHL, were examined in the present study (Supplemental Digital Content 1).

The follow-up was recorded and evaluated until August 2013. Patient data such as age and gender, clinical and functional examination, medical and surgical treatment were recorded and the treatment of hearing loss in the case of ISSNHL compared to NISSNHL was evaluated.

The extent of the initial hearing loss was described using the pure-tone average (PTA) in decibels hearing level (dB HL). The average hearing loss of the three most affected frequencies (3PTAmax), 10 frequencies (10PTA: 0.125; 0.25; 0.5; 1; 1.5; 2; 3; 4; 6; 8 kHz), 9 frequencies (9PTA: 0.125; 0.25; 0.5; 1; 2; 3; 4; 6; 8 kHz), 4frequencies (4PTA: 0.5; 1; 2; 4 kHz), low- (LF3PTA: 0.125; 0.5; 1 kHz), middle- (MF3PTA: 2; 3; 4 kHz), and high frequency (HF2PTA: 6; 8 kHz) hearing loss were calculated [3, 13, 14]. Hearing losses that were not technically measurable and deafness were considered as hearing loss of 120 dB. According to Plontke et al., the outcome was calculated as an absolute hearing improvement before therapy compared to the follow-up (ΔPTA_abs_ = PTA_pre_ minus PTA_post_ in dB) [13]. Furthermore, relative (rel) hearing improvement was calculated as ΔPTA_rel_ = 100*(PTA_pre_ minus PTA_post_)/PTA_pre_ and relative hearing improvement compared to the contralateral ear (contral) ΔPTA_relcontral_ = 100* (PTA_pre_ minus PTA_post_)/(PTA_pre_ minus PTA _contral_). ΔPTA_abs_ ≥ 10 dB, ΔPTA_abs_ ≥ 15 dB, ΔPTA_rel_ ≥ 50% and ΔPTA_relcontral_ ≥ 50% in a dichotomous distribution (yes / no) were considered as criterions for a successful improvement [13]. Kanazaki et al. defines no recovery as < 10 dB hearing improvement relative to the initial hearing loss. Each hearing gain of  ≥ 10 dB is defined as at least partial hearing gain, which is why an absolute hearing gain of ≥ 10 dB was considered as a criterion for success in the univariate analysis in this study [15]. As the endpoint of the univariate analyses, we used the 3PTAmax as it was done before [3, 14, 16].

The epidemiological statistics were calculated on the basis of the annual average population of Thuringia from 2011 and 2012, which are published in the online database of the statistical office of the federal state of Thuringia (www.tls.thueringen.de).

The patients affected by ISSNHL were treated according to the German guidelines for the treatment of sudden hearing loss: All patients received intravenous prednisolone therapy. Prednisolone was administered in a dose of 250 mg/d (range of 100–500 mg/d) [7, 8]. The dose was then reduced over 7 to 10 days. If there was no improvement in hearing within 3 days under prednisolone therapy and the hearing was below 80 dB in 4PTA, tympanoscopy with round window membrane sealing was performed. If the hearing threshold 4PTA after 3 days of prednisolone treatment was still below 40 dB salvage intratympanic dexamethasone instillation was performed [7]. There was no standardized procedure for performing dexamethasone instillation. In addition to the specific treatment of the cause of their hearing loss, patients with NISSNHL received also a therapy with prednisolone according to the above-mentioned scheme. Patients with varicella zoster infection were treated with acyclovir. Patients with acute otitis media received antibiotic therapy and paracentesis. Patients with Lyme disease received doxycycline or ceftriaxone. Patients with Menière disease were treated with glucocorticoids and antivertiginous therapy.

### Statistical analysis

Unless otherwise noted, data were presented with mean values ± standard deviation (SD). All statistical analyses were performed using IBM SPSS, version 24.0.0.0. The non-parametric Mann–Whitney-U-test for independent metric data was applied to compare different subgroups of patients. The Chi-square test was applied for independent nominal data. The non-parametric Wilcoxon test for dependent metric data was applied to analyze differences between initial hearing loss and final hearing loss on the affected ear at the end of the follow-up. A multivariate binary logistic regression was performed including the significant associations. Nominal p-values of two-tailed tests are reported. The significance level was set at *p* < 0.05.

## Results

### Subjects and treatment

**Fig. 1:**
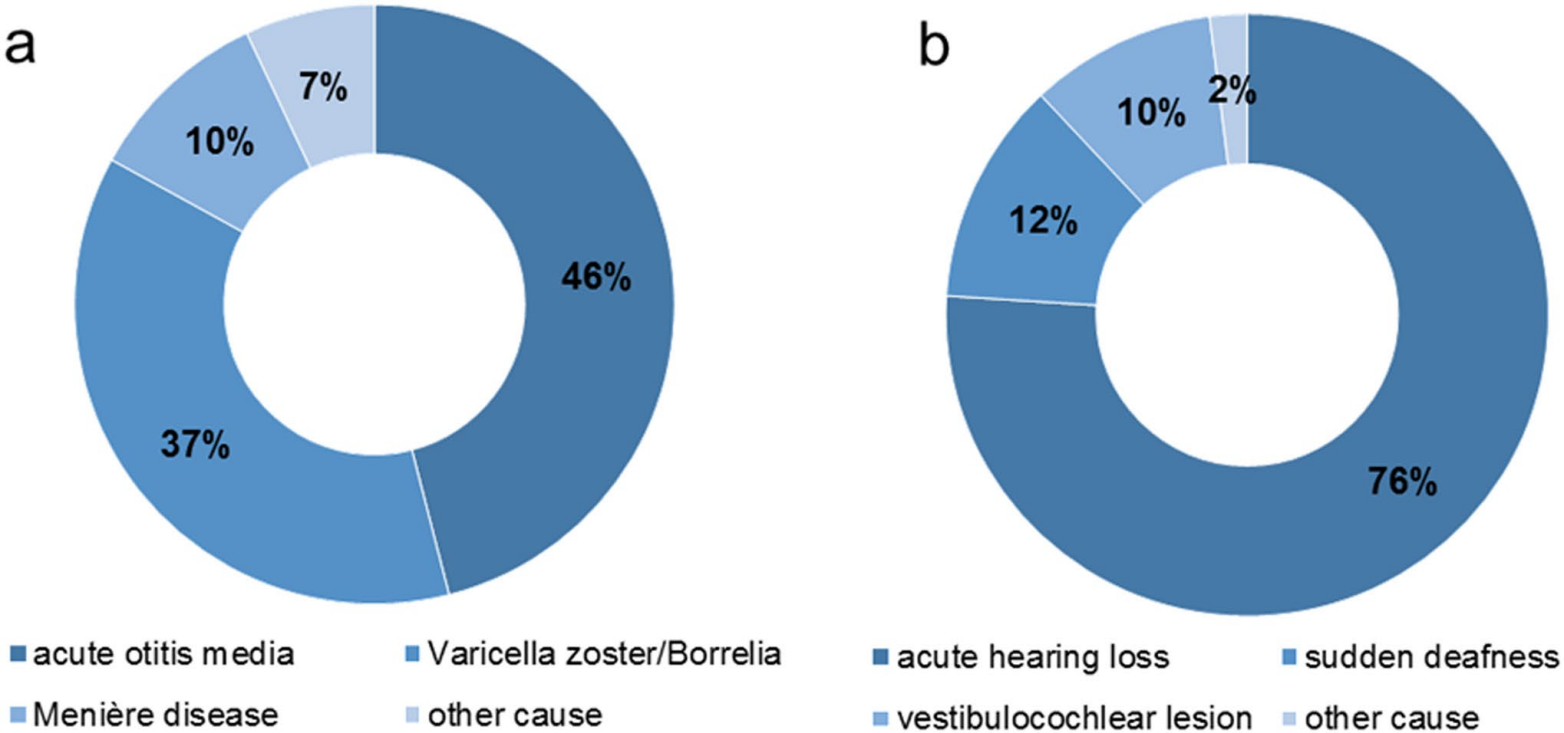
Frequency of distribution of the diagnoses of **a** non-idiopathic sudden sensorineural hearing loss (NISSNHL) and **b** idiopathic sudden sensorineural hearing loss (ISSNHL)

In 2011 and 2012, a total of 574 patients who were hospitalized in Thuringia for acute hearing loss were included in this study. The mean age was 57.2 ± 16 years. 51% of the patients were male, 49% female. 490 patients had ISSNHL (51% male, 49% female, mean age 55.7 years ± 15.9 years). 12% of the patients had an acute deafness and 2% had a combined vestibulocochlear lesion (Fig. 1a). In the other 84 patients, i.e. in 14.6% of cases, an underlying cause for the sudden hearing loss could be found. They were included in NISSNHL-group (54% male, 46% female, mean age 58.7 ± 15.8 years). 46% had acute otitis media, 37% had an acute infection with varicella zoster or Borrelia and 10% had Menière disease (Fig. 1b). The gender distribution was the same in both groups (*p* = 0.218). There was no side predominance of acute hearing loss neither in ISSNHL nor in NISSNHL (*p* = 0.197). The accompanying symptoms like tinnitus (ISSNHL: 62%; NISSNHL 51%; *p* = 0.743) or vertigo (ISSNHL: 30%; NISSNHL 32%; *p* = 0.605) occurred equally frequently in both groups (Table 1). There was no significant difference in the patients´ comorbidities either. Nicotine abuse (*p* = 0.117), coronary heart disease (*p* = 0.601), diabetes mellitus type II (*p* = 0.414), hypercholesterolemia (*p* = 0.755) and arterial hypertension (*p* = 0.754) occurred equally frequently in ISSNHL and NISSNHL (Table 2). More patients with NISSNHL (45%) than with ISSNHL (35%) received a prior outpatient treatment before admission to the hospital (*p* < 0.001). The time from the onset of hearing loss to hospital admission was less for NISSNHL (7.7 days ± 12.2 days) than for ISSNHL (8.9 days ± 11.8 days; *p* = 0.02). The majority of the patients (98.1%) received prednisolone therapy during the inpatient stay (100% in the NISSNHL group vs. 97.8% in the ISSNHL group; *p* = 0.166). Patients with NISSNHL had to undergo surgery during the hospital stay more often (52%) than patients with ISSNHL (29%; *p* < 0.001) (Table 3).

**Table 1:** Baseline characteristics and symptoms of patients with idiopathic sudden sensorineural hearing loss (ISSNHL) and patients with non-idiopathic sudden sensorineural hearing loss (NISSNHL)

Patients´ characteristics	All patients		NISSNHL		ISSNHL		
	*N*	%	*N*	%	*N*	*%*	*p*
Gender							
Male	294	51.2	45	53.6	251	50.8	0.218
Female	280	48.8	39	46.6	239	49.2	
Side							
Right	277	48.3	46	54.8	231	47.1	0.197
Left	297	51.7	38	45.2	259	52.9	
Tinnitus							
Yes	351	61.1	49	51.4	302	61.6	0.743
No	214	37.3	33	31.3	181	36.9	
n.a	9	1.6	2	2.4	7	1.4	
Vertigo							
Yes	175	30.5	27	32.1	148	30.2	0.605
No	396	69	56	66.7	340	69.4	
n.a	3	0.5	1	1.2	2	0.4	
	Mean	SD	Mean	SD	Mean	SD	
Age	57.2	16.0	55.7	15.9	58.7	15.8	0.100

*n.a*. not available, *SD* standard deviation

**Table 2:** Comorbidities of patients with idiopathic sudden sensorineural hearing loss (ISSNHL) and patients with non-idiopathic sudden sensorineural hearing loss (NISSNHL)

Patients´ characteristics	All patients		NISSNHL		ISSNHL		
	*N*	%	*N*	%	*N*	%	*p*
Smoking							
Yes	93	16.2	20	23.8	73	14.9	0.117
No	471	82.1	63	75	408	83.3	
n.a	10	1.7	1	1.2	9	1.8	
Coronary heart disease							
Yes	68	11.5	11	13.1	57	11.6	0.601
No	503	87.6	72	85.7	431	88	
n.a	3	0.5	1	1.2	2	0.4	
Diabetes							
Yes	92	16	16	19	76	15.5	0.414
No	482	84	68	81	414	84.5	
Hypercholesterolemia							
Yes	76	13.2	13	15.5	63	12.9	0.755
No	493	85.9	70	83.3	423	86.3	
n.a	5	0.9	1	1.2	4	0.8	
Arterial hypertension							
Yes	319	55.6	48	57.1	271	55.3	0.754
No	255	44.4	36	42.9	219	44.7	
Comorbidity							
Yes	187	67.2	30	35.7	157	32	0.742
No	386	32.6	54	64.3	332	67.8	
n.a	1	0.2	0	0	1	0.2	

*n.a.* not available

**Table 3:** Therapy of patients with idiopathic sensorineural hearing loss (ISSNHL) and non-idiopathic sensorineural hearing loss (NISSNHL)

Parameters	All patients		NISSNHL		ISSNHL		*p*
	*N*	%	*N*	%	*N*	%	
Outpatient							
pretreatment							
Yes	210	36.6	38	45.2	172	35.1	**< 0.001**
No	362	63.1	44	52.4	318	64.9	
n.a	2	0.3	2	2.4	0	0	
Inpatient							
prednisolone							
treatment							
Yes	563	98.1	84	100	479	97.8	0.166
No	11	1.9	0	0	11	2.2	
Surgical Treatment							
Yes	186	32.4	44	52.4	142	29.0	**< 0.001**
No	388	67.6	40	47.6	348	71	
Interval onset of hearing loss to inpatient treatment	days	SD	days	SD			
			7.7	12.2	8.9	11.8	**0.020**

*n.a.* not available, Significant *p*-values (*p* < 0.05) in bold, *SD* standard deviation

### Hearing loss and recovery

The average initial hearing loss of the three most affected frequencies (3PTAmax) at NISSNHL was 67.4 dBHL ± 30.5dBHL and showed no statistical difference to hearing loss at ISSNHL with a 3PTAmax of 72.9 dBHL ± 31.3 dB (*p* = 0.124). Considering the 10PTA, 9PTA, 4PTA, LF-3PTA and MF-3PTA ISSNHL had more severe hearing loss than NISSNHL (*p* < 0.05) (Table 4). The pre- and post-treatment hearing level showed a significant improvement in 10PTA, 9PTA, 4PTA, LF-3PTA, MF-3PTA, HF-2PTA and 3PTAmax in NISSNHL and ISSNHL (Table 5). Under therapy, patients with NISSNHL improved by 10.2 dB ± 17.6 dB and patients with ISSNHL by 8.1 dB ± 18.8 dB considering the 3PTAmax_abs_. There was no statistically significant difference in NISSNHL and ISSNHL considering the ΔPTA_abs_, the ΔPTA_rel_ and the ΔPTA_relcontral_ in all endpoints (*p* > 0.05) (Table 5). If ΔPTA_abs_ ≥ 10 dB was used as the measure of successful hearing recovery, there was a significant difference in both groups: 48.8% of the NISSNHL, showed a hearing improvement of ≥ 10 dB in Δ3PTAmax_abs_, while for ISSNHL only 34.3% had a corresponding hearing improvement (*p* = 0.011). In LF-3PTA 27.4% of NISSNHL and 42% of ISSNHL reached ΔPTA_abs_ ≥ 10 dB (*p* = 0.011) and 17.9% of NISSNHL and 32.2% of ISSNHL reached ΔPTA_abs_ ≥ 15 dB. Other parameters showed no difference in NISSNHL and ISSNHL (Table 6).

**Table 4:** Mean hearing loss in all patients and in patients with idiopathic (ISSNHL) and non-idiopathic sudden sensorineural hearing loss (NISSNHL)

Parameter	all Mean (dBHL)	SD (dBHL)	ISSNHL Mean (dBHL)	SD (dBHL)	NISSNHL Mean (dBHL)	SD (dBHL)	*p*
10PTA, dBHL	56.4	31.4	58.0	31.7	47.0	28.1	**0.003**
9PTA, dBHL	56.6	31.1	58.2	31.4	47.6	27.9	**0.004**
4PTA, dBHL	55.9	32.9	57.8	33.1	44.5	29.4	**0.000**
LF-3PTA, dBHL	50.4	32.8	52.8	32.8	36.5	29.6	**0.000**
MF-3PTA, dBHL	59.4	34.2	60.8	34.6	51.2	30.6	**0.016**
HF-2PTA, dBHL	68.0	37.1	68.3	37.5	66.1	34.6	0.616
3PTAmax, dBHL	72.1	31.2	72.9	31.3	67.4	30.5	0.124

10PTA (0.125; 0.25; 0.5; 1; 1.5; 2; 3; 4; 6; 8 kHz), 9PTA (0.125; 0.25; 0.5; 1; 2; 3; 4; 6; 8 kHz), 4PTA: (0.5; 1; 2; 4 kHz), LF3PTA (0.125; 0.5; 1 kHz) MF3PTA (2; 3; 4 kHz), HF2PTA (6; 8 kHz), 3PTAmax (PTA of the three most affected frequencies)

**Table 5:** Pre- and post-treatment hearing level, overall absolute and relative hearing improvement on the affected ear in patients with idiopathic and non-idiopathic sensorineural hearing loss

Parameter	Pre-treatment		Post-treatment			Absolute hearing gain, ΔPTA_abs_			Relative hearing gain, ΔPTA_rel_			Relative hearing gain contralateral, ΔPTA_relcontral_		
	Mean (dBHL)	SD (dBHL)	Mean (dBHL)	SD (dBHL)	*p*	Mean (dB)	SD (dB)	*p*	Mean (%)	SD (%)	*p*	Mean (%)	SD (%)	*p*
10PTA, dBHL														
ISSNHL	58.0	31.7	51.2	36.0	** < 0.001**	6.8	16.3	0.881	14.1	30.3	0.408	17.2	150.3	0.089
NISSNHL	47.0	28.1	40.4	30.3	** < 0.001**	6.7	16.8		14.8	32.3		88.0	545.6	
9PTA, dBHL														
ISSNHL	58.2	31.4	51.5	35.7	** < 0.001**	6.7	16.3	0.797	13.8	30.0	0.409	17.4	141.6	0.099
NISSNHL	47.6	27.9	41.0	30.3	** < 0.001**	6.6	16.8		14.7	32.1		82.1	488.0	
4PTA, dBHL														
ISSNHL	57.8	33.1	50.6	37.2	** < 0.001**	7.2	17.0	0.791	13.3	43.5	0.544	20.6	121.9	0.076
NISSNHL	44.5	29.4	38.0	31.6	** < 0.001**	6.5	17.9		13.8	44.3		31.1	103.7	
LF-3PTA, dBHL														
ISSNHL	52.8	32.8	43.8	36.4	**0.001**	9.0	18.7	0.051	17.6	43.5	0.563	27.6	76.8	0.564
NISSNHL	36.5	29.6	31.4	30.5	**0.001**	5.1	18.9		5.6	79.3		6.2	289.9	
MF-3PTA, dBHL														
ISSNHL	60.8	34.6	54.9	38.8	** < 0.001**	5.9	17.8	0.274	11.0	35.1	0.135	31.3	145.7	0.172
NISSNHL	51.2	30.6	43.2	32.1	** < 0.001**	8.0	18.9		16.0	34.3		22.7	131.4	
HF-2PTA, dBHL														
ISSNHL	68.3	37.5	63.6	39.5	**0.001**	4.6	23.5	0.134	6.6	33.6	0.081	15.3	121.5	0.145
NISSNHL	66.1	34.6	58.9	37.9	**0.001**	7.2	18.9		12.5	27.1		12.7	88.4	
3PTAmax dBHL														
ISSNHL	72.9	31.3	65.2	34.5	** < 0.001**	8.1	18.8	0.152	12.3	29.1	0.131			
NISSNHL	67.4	30.5	57.2	31.6	** < 0.001**	10.2	17.6		15.4	25.9				

10PTA (0.125; 0.25; 0.5; 1; 1.5; 2; 3; 4; 6; 8 kHz), 9PTA (0.125; 0.25; 0.5; 1; 2; 3; 4; 6; 8 kHz), 4PTA: (0.5; 1; 2; 4 kHz), LF3PTA (0.125; 0.5; 1 kHz) MF3PTA (2; 3; 4 kHz), HF2PTA (6; 8 kHz), 3PTAmax (PTA of the three most affected frequencies), ΔPTA_abs_ (Absolute hearing gain), ΔPTA_rel_ (Relative hearing gain), ΔPTA_relcontral_ (Relative hearing gain contralateral)

**Table 6:** Hearing recovery rates in patients with idiopathic and non-idiopathic sensorineural hearing loss

Parameter	ISSNHL (*n* = 490)	NISSNHL (*n* = 84)	*p*
10PTA, dBHL			
ΔPTAabs ≥ 10 dB (%)	34.9	28.6	0.258
ΔPTAabs ≥ 15 dB (%)	24.1	15.5	0.083
ΔPTArel ≥ 50% (%)	13.9	9.5	0.277
ΔPTArelcontral ≥ 50% (%)	29.2	33.3	0.443
9PTA, dBHL			
ΔPTAabs ≥ 10 dB (%)	34.7	27.4	0.190
ΔPTAabs ≥ 15 dB (%)	23.1	16.7	0.192
ΔPTArel ≥ 50% (%)	13.7	9.5	0.298
ΔPTArelcontral ≥ 50% (%)	30	33.3	0.540
4PTA, dBHL			
ΔPTAabs ≥ 10 dB (%)	36.7	29.8	0.218
ΔPTAabs ≥ 15 dB (%)	25.5	19	0.204
ΔPTArel ≥ 50% (%)	15.7	9.5	0.140
ΔPTArelcontral ≥ 50% (%)	30.2	39.3	0.098
LF-3PTA, dBHL			
ΔPTAabs ≥ 10 dB (%)	42	27.4	**0.011**
ΔPTAabs ≥ 15 dB (%)	32.2	17.9	**0.008**
ΔPTArel ≥ 50% (%)	21	17.9	0.508
ΔPTArelcontral ≥ 50% (%)	32.7	38.1	0.329
MF-3PTA, dBHL			
ΔPTAabs ≥ 10 dB (%)	29.4	33.3	0.466
ΔPTAabs ≥ 15 dB (%)	22.4	23.8	0.783
ΔPTArel ≥ 50% (%)	12.9	14.3	0.720
ΔPTArelcontral ≥ 50% (%)	28.8	38.1	0.086
HF-2PTA, dBHL			
ΔPTAabs ≥ 10 dB (%)	28	38.1	0.060
ΔPTAabs ≥ 15 dB (%)	20.4	21.4	0.831
ΔPTArel ≥ 50% (%)	10.2	9.5	0.849
ΔPTArelcontral ≥ 50% (%)	26.9	28.6	0.756
3PTAmax, dBHL			
ΔPTAabs ≥ 10 dB (%)	34.3	48.8	**0.011**
ΔPTAabs ≥ 15 dB (%)	25.9	28.6	0.610
ΔPTArel ≥ 50% (%)	10.2	8.3	0.597

10PTA (0.125; 0.25; 0.5; 1; 1.5; 2; 3; 4; 6; 8 kHz), 9PTA (0.125; 0.25; 0.5; 1; 2; 3; 4; 6; 8 kHz), 4PTA: (0.5; 1; 2; 4 kHz), LF3PTA (0.125; 0.5; 1 kHz) MF3PTA (2; 3; 4 kHz), HF2PTA (6; 8 kHz), 3PTAmax (PTA of the three most affected frequencies), ΔPTA_abs_ (Absolute hearing gain), ΔPTA_rel_ (Relative hearing gain), ΔPTA_relcontral_ (Relative hearing gain contralateral)

The subgroup analysis of patients with NISSNHL showed that patients with acute otitis media with 3PTAmax of 59.7 dBHL ± 24.6 dBHL and Menière disease with 3PTAmax of 50.4 dBHL ± 12.04 dBHL had a significantly lower initial hearing loss than the other subgroups (*p* = 0.033) (Table 7). The pre- and post-treatment hearing level showed significant differences for 3PTAmax in all subgroups. However, ΔPTA_abs_, ΔPTA_rel_ and ΔPTA_relcontral_ showed no differences between the subgroups (**Table ****8**). If ΔPTArel ≥ 50% and ΔPTA_relcontral_ ≥ 50% was used as success criteria for a hearing recovery, it could be seen that the same number of patients from all subgroups met the criterion. For ΔPTA_abs_ ≥ 10 dB and ΔPTA_abs_ ≥ 15 dB we could show a difference in the hearing recovery rate in between the subgroups for LF-3PTA. 12,8% of patient with acute otitis media, 45,2% of patients with varicella zoster or Borrelia, 25% of patients with Menière disease and 16.7% others met the ΔPTA_abs_ ≥ 10 dB for the LF-3PTA (*p* = 0.021) (Table 9).

**Table 7:** Mean hearing loss in subgroups of non-idiopathic sudden sensorineural hearing loss (NISSNHL)

Parameter	AOM Mean (dBHL)	SD (dBHL)	VZB Mean (dBHL)	SD (dBHL)	M Mean (dBHL)	SD (dBHL)	O Mean (dBHL)	SD (dBHL)	p
10PTA, dBHL	36.9	16.5	62.5	34.7	32.7	9.3	52.3	32.0	**0.007**
9PTA, dBHL	37.7	16.8	62.7	34.4	33.8	9.4	52.8	32.0	**0.010**
4PTA, dBHL	32.8	14.5	62.4	36.6	27.5	9.5	50.6	31.2	**0.001**
LF-3PTA, dB HL	21.5	13.9	55.8	35.7	33.5	7.9	38.9	35.5	**0.000**
MF-3PTA, dBHL	43.1	17.9	66.3	37.8	25.4	15.0	60.6	32.8	**0.002**
HF-2PTA, dBHL	62.3	29.8	75.6	38.7	41.3	18.8	75.4	43.1	0.062
3PTAmax dBHL	59.7	24.6	80.1	34.2	50.4	12.0	74.7	40.2	**0.033**

*AOM* Acute otitis media, *VZB* Varicella zoster/Borrelia, *M* Menière disease, *O* Other cause, 10PTA (0.125; 0.25; 0.5; 1; 1.5; 2; 3; 4; 6; 8 kHz), 9PTA (0.125; 0.25; 0.5; 1; 2; 3; 4; 6; 8 kHz), 4PTA: (0.5; 1; 2; 4 kHz), LF3PTA (0.125; 0.5; 1 kHz) MF3PTA (2; 3; 4 kHz), HF2PTA (6; 8 kHz), 3PTAmax (PTA of the three most affected frequencies)

**Table 8:** Pre- and post-treatment hearing level, overall absolute and relative hearing improvement on the affected ear in subgroups of patients with non-idiopathic sensorineural hearing loss

Parameter	Pre-treatment		Post-treatment			Absolute hearing gain, ΔPTA_abs_			Relative hearing gain, ΔPTA_rel_			Relative hearing gain contralateral, ΔPTA_relcontral_		
	Mean (dBHL)	SD (dBHL)	Mean (dBHL)	Mean (dBHL)	*p*	Mean (dB)	SD (dB)	*p*	Mean (%)	*SD (%)*	*p*	Mean (%)	SD (%)	*p*
10PTA, dBHL														
AOM	36.9	16.5	29.9	15.0	** < 0.001**	7.0	8.6	0.796	18.9	21.3	0.336	160.6	796.5	0.526
VZB	62.5	34.7	54.6	38.9	**0.044**	7.9	24.7		11.2	42.4		24.7	75.6	
MD	32.7	9.3	27.1	14.7	0.161	5.6	10.1		21.7	35.8		40.9	64.9	
O	52.3	32.0	65.9	42.1	0.114	3.4	15.5		− 2.3	24.5		5.7	46.1	
9PTA, dBHL														
AOM	37.7	16.8	30.7	15.2	** < 0.001**	7.0	8.7	0.783	18.5	21.3	0.292	145.8	711.3	0.565
VZB	62.7	34.4	55.1	38.7	**0.056**	7.7	24.8		11.2	42.1		26.9	81.8	
MD	33.8	9.4	27.6	14.9	0.123	6.3	10.3		22.7	35.5		42.2	64.6	
O	52.8	32.0	66.1	41.9	0.138	3.2	15.6		− 2.4	24.1		5.4	48.0	
4PTA, dBHL														
AOM	32.8	14.5	25.9	13.1	** < 0.001**	6.9	8.3	0.691	20.6	22.3	0.442	50.7	80.0	0.380
VZB	62.4	36.6	54.4	40.8	0.118	8.0	26.5		6.4	62.2		10.3	137.3	
MD	27.5	9.5	24.4	15.8	0.483	3.1	12.7		19.0	52.8		31.6	79.0	
O	50.6	31.2	65.0	43.3	0.073	4.5	16.2		0.5	26.8		10.4	44.4	
LF-3PTA, dBHL														
AOM	21.5	13.9	18.5	12.3	**0.005**	3.0	8.0	0.515	5.1	77.2	0.770	40.9	180.8	0.912
VZB	55.8	35.7	46.5	38.8	**0.034**	9.2	28.3		5.5	95.1		− 39.9	424.1	
MD	33.5	7.9	29.8	17.4	0.484	3.8	15.4		13.4	51.3		23.0	79.0	
O	38.9	35.5	58.1	43.5	**0.020**	5.3	17.3		− 2.0	37.3		13.6	74.0	
MF-3PTA, dBHL														
AOM	43.1	17.9	33.8	17.0	** < 0.001**	9.3	10.6	0.28	21.5	23.8	0.284	45.4	64.0	0.301
VZB	66.3	37.8	58.1	41.1	0.189	8.2	26.2		9.8	42.9		− 10.6	196.5	
MD	25.4	15.0	20.6	12.9	0.310	4.8	11.9		23.8	42.0		49.1	82.1	
O	60.6	32.8	69.8	44.1	0.638	3.3	19.1		0.8	30.5		12.4	48.8	
HF-2PTA, dBHL														
AOM	62.3	29.8	52.2	28.9	** < 0.001**	10.1	17.0	0.093	15.6	22.9	0.097	14.2	95.8	0.577
VZB	75.6	38.7	70.6	44.8	0.502	5.0	23.4		8.5	34.1		18.3	53.7	
MD	41.3	18.8	32.8	16.6	**0.017**	8.4	9.0		22.0	21.1		− 12.6	162.9	
O	75.4	43.1	78.6	43.8	0.602	0.6	17.6		− 0.8	11.1		11.2	44.0	
3PTAmax, dBHL														
AOM	59.7	24.6	49.5	23.4	** < 0.001**	10.2	13.3	0.671	16.9	20.6	0.324			
VZM	80.1	34.2	69.2	37.6	**0.003**	10.9	23.6		13.5	31.4				
MD	50.4	12.0	38.3	17.7	**0.042**	12.1	14.3		25.2	31.5				
O	74.7	40.2	76.2	37.6	**0.016**	5.6	15.6		2.0	13.1				

*AOM* Acute otitis media, *VZB* Varicella zoster/Borrelia, *M*Menière disease, *O* Other cause, 10PTA (0.125; 0.25; 0.5; 1; 1.5; 2; 3; 4; 6; 8 kHz), 9PTA (0.125; 0.25; 0.5; 1; 2; 3; 4; 6; 8 kHz), 4PTA: (0.5; 1; 2; 4 kHz), LF3PTA (0.125; 0.5; 1 kHz) MF3PTA (2; 3; 4 kHz), HF2PTA (6; 8 kHz), 3PTAmax (PTA of the three most affected frequencies), ΔPTA_abs_ (Absolute hearing gain), ΔPTA_rel_ (Relative hearing gain), ΔPTA_relcontral_ (Relative hearing gain contralateral)

**Table 9:** Hearing recovery rates in subgroups of pathients with non-idiopathic sensorineural hearing loss

Parameter	AOM	VZB	M	O	p
10PTA, dBHL					
ΔPTAabs ≥ 10 dB (%)	23.1	35.5	37.5	16.7	0.571
ΔPTAabs ≥ 15 dB (%)	10.3	19.4	25	16.7	0.635
ΔPTArel ≥ 50% (%)	7.7	9.7	25	0	0.396
ΔPTArelcontral ≥ 50% (%)	33.3	35.5	37.5	16.7	0.833
9PTA, dBHL					
ΔPTAabs ≥ 10 dB (%)	23.1	32.3	25	16.7	0.688
ΔPTAabs ≥ 15 dB (%)	12.8	19.4	25	16.7	0.810
ΔPTArel ≥ 50% (%)	7.7	9.7	25	0	0.395
ΔPTArelcontral ≥ 50% (%)	35.9	32.3	37.5	16.7	0.818
4PTA, dBHL					
ΔPTAabs ≥ 10 dB (%)	28.2	35.5	37.5	16.7	0.778
ΔPTAabs ≥ 15 dB (%)	15.4	22.6	25	16.7	0.853
ΔPTArel ≥ 50% (%)	7.7	9.7	25	0	0.396
ΔPTArelcontral ≥ 50% (%)	46.2	32.3	50	16.7	0.384
LF-3PTA, dBHL					
ΔPTAabs ≥ 10 dB (%)	12.8	45.2	25	16.7	**0.021**
ΔPTAabs ≥ 15 dB (%)	5.1	32.3	37.5	0	**0.008**
ΔPTArel ≥ 50% (%)	15.4	22.6	25	0	0.534
ΔPTArelcontral ≥ 50% (%)	38.5	41.9	25	33.3	0.843
MF-3PTA, dBHL					
ΔPTAabs ≥ 10 dB (%)	35.9	35.5	25	16.7	0.761
ΔPTAabs ≥ 15 dB (%)	25.6	25.8	12.5	16.7	0.834
ΔPTArel ≥ 50% (%)	17.9	6.5	37.5	0	0.093
ΔPTArelcontral ≥ 50% (%)	41	35.5	25	33.3	0.962
HF-2PTA, dBHL					
ΔPTAabs ≥ 10 dB (%)	41	35.5	50	16.7	0.602
ΔPTAabs ≥ 15 dB (%)	28.2	19.4	12.5	16.7	0.816
ΔPTArel ≥ 50% (%)	5.1	12.9	25	0	0.252
ΔPTArelcontral ≥ 50% (%)	30.8	25.8	37.5	16.7	0.819
3PTAmax, dBHL					
ΔPTAabs ≥ 10 dB (%)	51.3	48.4	50	33.3	0.880
ΔPTAabs ≥ 15 dB (%)	7.7	25.8	50	16.7	0.511
ΔPTArel ≥ 50% (%)	5.1	9.7	25	0	0.261

AOM: Acute otitis media, VZB: Varicella zoster/Borrelia, M:Menière disease, O: Other cause, 10PTA (0.125; 0.25; 0.5; 1; 1.5; 2; 3; 4; 6; 8 kHz), 9PTA (0.125; 0.25; 0.5; 1; 2; 3; 4; 6; 8 kHz), 4PTA: (0.5; 1; 2; 4 kHz), LF3PTA (0.125; 0.5; 1 kHz) MF3PTA (2; 3; 4 kHz), HF2PTA (6; 8 kHz), 3PTAmax (PTA of the three most affected frequencies), ΔPTA_abs_ (Absolute hearing gain), ΔPTA_rel_ (Relative hearing gain), ΔPTA_relcontral_ (Relative hearing gain contralateral)

The univariate analysis of the prognostic factors showed that among the patients with NISSNHL patients without vertigo more often had a successful hearing impairment Δ3PTAmax_abs_ ≥ 10 dB (*p* = 0.027) and that more patients with prior outpatient treatment showed a hearing impairment of Δ3PTAmax_abs_ ≥ 10 dB (*p* = 0.032). There was no difference for the tinnitus (*p* = 0.325), as well as for the comorbidities of coronary heart disease (*p* = 0.531), hypercholesterolemia (*p* = 0.439), diabetes mellitus type II (*p* = 0.653) and arterial hypertension (*p* = 0.850) (Table 10).

**Table 10:** Univariate association between patients’ and treatment characteristics versus a successful recovery defined as Δ3PTAmax_abs_ ≥ 10 dB (*N* = 84)

Parameter	Δ3PTAmax_abs_˂10 dB	Δ3PTAmax_abs_ ≥ 10 dB	*p*
Patients´characteristics			
Gender			0.083
Male	27	18	
Female	16	23	
Side			0.524
Right	25	21	
Left	18	20	
Tinnitus			0.325
Yes	25	24	
No	16	17	
n.a	2	0	
Vertigo			**0.027**
Yes	19	8	
No	23	33	
n.a	1	0	
Smoking			0.557
Yes	11	9	
No	32	31	
n.a	0	1	
Coronary heart disease			0.531
Yes	5	6	
No	38	34	
n.a	0	1	
Diabetes mellitus type II			0.653
Yes	9	7	
No	34	34	
Hypercholesterolemia			0.439
Yes	8	5	
No	35	35	
n.a	0	1	
Arterial hypertension			0.850
Yes	25	23	
No	18	18	
Comorbidity			0.454
Yes	17	13	
No	26	23	
Prior outpatient treatment			**0.032**
Yes	14	24	
No	27	17	
n.a	2	0	
Surgical treatment			0.835
Yes	23	21	
No	20	20	

3PTAmax (PTA of the three most affected frequencies), ΔPTA_abs_ (Absolute hearing gain)

The multivariate analysis showed that neither vertigo nor prior outpatient treatment were independent factors associated with better hearing recovery (Table 11).

**Table 11:** Multivariate binary regression of predictors of successful improvement of hearing Δ3PTAmax_abs_ ≥ 10 dB

Parameter	B	95% CI lower	95% CI upper	*p*
Vertigo	0.009	− 0.096	0.114	0.865
Prior outpatient treatment	− 0.001	0.397	− 0.002	0.397

*CI* confidence interval

### Epidemiology

Thuringia had an average of 2,176,031 (female: 1,105,434, male: 1,070,597) inhabitants in 2011 and 2012. In total, an average of 16.61 inpatients per 100,000 habitants was treated in Thuringia per year. Of these, 11.25 inpatients per 100,000 habitants had an ISSNHL and 1.93 per 100,000 inhabitant people per year had an NISSNHL (0.89/100,000 for acute otitis media, 0.71/100,000 for varicella zoster or Borrelia and 0.18/100,000 for Menière disease). There was no difference in gender distribution. The incidence of the ISSNHL was 21.6/100,000 in women and 23.4/100,000 in men. The incidence of NISSNHL was 3.5/100,000 in women and 4.2/100,000 in men.

## Discussion

### Key findings

In the current study, causes for sensorineural hearing losses were found in 14.6%, which corresponds to the numbers reported so far [7]. There were no differences between patients with ISSNHL and NISSNHL in terms of risk factors and accompanying symptoms. The extent of the initial absolute hearing loss tended to be higher in patients with ISSNHL compared to NISSNHL but the absolute and relative hearing recovery showed no difference. If according to Plontke et al. an absolute improvement of the pure tone average by ≥ 10 dB is used as a criterion for a successful hearing recovery, it can be seen that patients with NISSNHL show a successful hearing recovery more often than patients with ISSNHL considering the Δ3PTAmax_abs_ [13]. An explanatory model offers the possibility of using specific therapy options in the case of NISSNHL (acyclovir, antibiotics) [17, 18], while at ISSNHL therapy decisions are made without knowledge of the etiology of the hearing loss [7].

### Strength and limitations

Studies that directly compare ISSNHL and NISSNHL are not known. Therefore, the retrospective study presented here with a total of 490 patients with ISSNHL and 84 patients with NISSNHL is the largest study of this type published to date. One disadvantage of the current study is that only patients with acute hearing loss were included here. Patients treated in hospital with the ICD codes H65.0, H65.1, H65.2, H65.3 H65.4 H65.9, H66.0, H66.1, H66.2, H66.3, H66.4, H66.9, H67.0* H67.1*, H67.8*, H83.0, H73.0, J11.8,H70.0, H70.2, H70 0.8, H70.9, B02.8 and A69.2 for any underlying disease were not included. Therefore, there is an unreported number of patients with other diseases combined with sensorineural hearing loss. Likewise, the true incidence of diseases is underestimated here because only patients who have been hospitalized are included in this evaluation. This is associated with a selection bias in favor of the more severe cases. The evaluation of hearing loss and hearing gain is handled inconsistently in most studies [19–21]. So far, there is no consensus on the evaluation of hearing loss and hearing recovery in the pure tone audiogram [13]. The different criteria for evaluating the hearing loss and hearing gain make it difficult to compare studies with one another. In addition, the evaluation of different endpoints in this analysis also shows different results.

### Comparison with other studies

The data on the occurrence, extent and recovery of ISSNHL have already been discussed in detail elsewhere [3]. Therefore, we now focus on the data of NISSNHL. The incidence of acute otitis media is 10.85% [22]. The incidence of zoster oticus is 5 / 100,000 inhabitants [23]. The incidence of Lyme disease is 0.04 / 100,000 inhabitants, which is strongly dependent on the region [24]. Menière disease has an incidence of 200 / 100,000 inhabitants [25]. The epidemiological data diverge greatly in the evaluation published here. The reason might be that, as mentioned before, ICD-codes for underlying illnesses of hearing loss are not included here.

In addition to potentially life-threatening complications, acute otitis media can lead to a permanent impairment of the patient due to hearing loss [22]. A zoster oticus can lead to accompanying facial palsy or vestibular failure in the context of Ramsay Hunt syndrome [20, 26]. Overall, the detection of Borrelia titers is controversial in the diagnosis of acute hearing loss. Numerous studies have shown a connection between Borrelia detection and sudden hearing loss, while others see no connection [27–32]. In Menière disease, hearing loss is one of the diagnostic criteria of the Bárány Society and the AAO-HNS guideline [33, 34]. It is noticeable that hearing impairment has different values in the underlying diseases. While in acute otitis media there is a complicated course in the case of sensorineural hearing loss, the detection of at least one episode of sensorineural hearing loss is a prerequisite for the diagnosis of Menière disease.

In the present study, it was found that acute otitis media and Menière disease showed significantly less absolute hearing loss compared to the other subgroups. Many evaluations consider hearing loss, but often no distinction is made between the appearance of conductive hearing loss and sensorineural hearing loss. Occasionally the absolute extent of hearing loss (in dBHL) is not described. For acute otitis media hearing loss is reported between 25 and 40 dBHL [19, 22, 35–37]. Hearing loss in zoster oticus is reported in 7–85% of patients with an extend of 10dBHL to 20dBHL [20, 21, 38–40], while hearing loss in case of Borrelia infection is considered in approximately 12% [41]. The largest clinical trial examining Menière disease includes 350 patients. This showed fluctuating curves in the pure tone audiogram at the beginning of the disease and an average hearing threshold of 26 to 40 dBHL[42]. In summary, one subgroup in this study showed a higher absolute hearing loss in the used endpoints (Varicella / Borrelia: range 55.8dBHL – 80.1dBHL), whereas initial PTA of the others (acute otitis media: range 21.5dBHL – 59.7dBHL, Menière disease range: 25.4—50.42 dBHL) considering the different endpoints is the same as reported in the underlying literature. Explanations might be the already mentioned bias to more severe cases and difficulties in comparison of different studies reporting a hearing loss.

So far, there is no guideline for the treatment of acute otitis media with sensorineural hearing loss in Germany. The German Society for General Medicine and Family Medicine published a S2k-guideline "Earache": In the guideline, initially symptomatic treatment and, in the event of a lack of improvement or indications of a complicated course, antibiotics are used [43]. In acute otitis media, the patients in the studies considered were treated with oral antibiotics [19, 36]. Oral corticosteroids and paracentesis with or without tympanic drainage were optionally performed [19]. Patients with herpes zoster oticus were treated intravenously with acyclovir [44]. There is a German S2k-guideline in which anti-viral therapy in combination with glucocorticoid therapy is recommended for zoster oticus, but this guideline does not give specific recommendations regarding a related sensorineural hearing loss [45]. Patients with Lyme disease are treated with ceftriaxone or doxycycline depending on the stage of the disease [41]. The treatment strategies in the current study thus corresponded to current treatment recommendations. In 87.5% of cases with acute otitis media and sensorineural hearing loss there was an improvement in hearing of at least 10 dB on average with 5PTA (500 Hz, 1 kHz, 2 kHz, 3 kHz, 4 kHz) [19]. In the current evaluation, there was no difference in hearing improvement after therapy in between all subgroups.

## Conclusion

The current retrospective study examines inpatients with ISSNHL and NISSNHL in Thuringia in 2011 and 2012. It can be seen that ISSNHL tends to have a higher initial hearing loss than patients with NISSNHL. ISSNHL and NISSNHL show no difference in the degree of absolute or relative hearing improvement. However, patients with NISSNHL are more likely to show successful hearing improvement considering the 3PTAmax. The data are not sufficient to show prognostic differences of subgroups at NISSNHL. However, we were able to show that patients with acute otitis media and Menière disease show an initially lower hearing loss compared to patients with varicella zoster or Lyme disease or other underlying diseases.

## Supplementary Information

Below is the link to the electronic supplementary material.

Supplementary file 1 (TIF 12771 KB) Supplemental Digital Content 1. Inclusion and exclusion of patients´ datasets. 574 patients were analyzed. 490 patients had an ISSNHL (idiopathic sudden sensorineural hearing loss), 84 patients had a NISSNHL (non-idiopathic sudden sensorineural hearing loss).

## Data Availability

All authors had full access to all of the data in the study. JT takes responsibility for the integrity of the data and the accuracy of the data analysis. No additional data are available.
